# Structure-revealing data fusion

**DOI:** 10.1186/1471-2105-15-239

**Published:** 2014-07-12

**Authors:** Evrim Acar, Evangelos E Papalexakis, Gözde Gürdeniz, Morten A Rasmussen, Anders J Lawaetz, Mathias Nilsson, Rasmus Bro

**Affiliations:** Department of Food Science, Faculty of Science, University of Copenhagen, Frederiksberg C, Denmark; School of Computer Science, Carnegie Mellon University, Pittsburgh, PA USA; Department of Nutrition, Exercise and Sports, Faculty of Science, University of Copenhagen, Frederiksberg C, Denmark; School of Chemistry, University of Manchester, Oxford Road, Manchester, M13 9PL UK

**Keywords:** Data fusion, Coupled matrix and tensor factorizations, Optimization, Sparsity, NMR, DOSY, MS

## Abstract

**Background:**

Analysis of data from multiple sources has the potential to enhance knowledge discovery by capturing underlying structures, which are, otherwise, difficult to extract. Fusing data from multiple sources has already proved useful in many applications in social network analysis, signal processing and bioinformatics. However, data fusion is challenging since data from multiple sources are often (i) heterogeneous (i.e., in the form of higher-order tensors and matrices), (ii) incomplete, and (iii) have both shared and unshared components. In order to address these challenges, in this paper, we introduce a novel unsupervised data fusion model based on joint factorization of matrices and higher-order tensors.

**Results:**

While the traditional formulation of coupled matrix and tensor factorizations modeling only shared factors fails to capture the underlying structures in the presence of both shared and unshared factors, the proposed data fusion model has the potential to automatically reveal shared and unshared components through modeling constraints. Using numerical experiments, we demonstrate the effectiveness of the proposed approach in terms of identifying shared and unshared components. Furthermore, we measure a set of mixtures with known chemical composition using both LC-MS (Liquid Chromatography - Mass Spectrometry) and NMR (Nuclear Magnetic Resonance) and demonstrate that the structure-revealing data fusion model can (i) successfully capture the chemicals in the mixtures and extract the relative concentrations of the chemicals accurately, (ii) provide promising results in terms of identifying shared and unshared chemicals, and (iii) reveal the relevant patterns in LC-MS by coupling with the diffusion NMR data.

**Conclusions:**

We have proposed a structure-revealing data fusion model that can jointly analyze heterogeneous, incomplete data sets with shared and unshared components and demonstrated its promising performance as well as potential limitations on both simulated and real data.

**Electronic supplementary material:**

The online version of this article (doi:10.1186/1471-2105-15-239) contains supplementary material, which is available to authorized users.

## Background

Data fusion, in other words, joint analysis of data from multiple sources, has proved useful in many disciplines. For instance, in bioinformatics, jointly analyzing multiple data sets representing different organisms [1, 2] or different tissue types [3, 4] improves the understanding of the underlying biological processes. Similarly, in metabolomics, biological fluids such as blood or urine, are investigated using different analytical techniques, e.g., LC-MS and NMR, and their fusion has the potential for more accurate biomarker identification [5–7].

**Figure 1:**
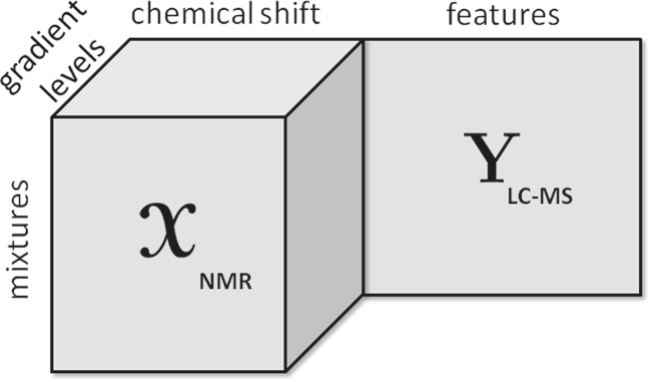
**A third-order tensor coupled with a matrix.**

An effective way of jointly analyzing data from multiple sources is to represent data from different sources as a collection of matrices, and then jointly analyze these matrices using collective matrix factorization [8]. Matrix factorization-based data fusion studies have been successfully applied in social network analysis [9, 10], signal processing [11, 12] and bioinformatics [1, 2, 4, 5, 13]. Recently, joint matrix factorization approaches have been extended to joint analysis of heterogeneous data sets, i.e., data in the form of matrices and higher-order tensors [14–17]. For instance, mixtures studied by NMR spectroscopy (a.k.a. DOSY - diffusion-ordered spectroscopy [18, 19]) can be represented as a third-order tensor with modes: mixtures, chemical shift and gradient levels [20, 21] while LC-MS measurements of the same mixtures can be represented using a mixtures by features matrix (see Figure 1). Joint factorization of such heterogeneous data has been studied to analyze multi-relational data, particularly, in social networks [15, 22–24].

While there are many successful applications of joint data analysis, the traditional formulation of joint factorization of multiple data sets is based on modeling only shared factors. However, data from multiple sources often have both shared and unshared components. If the goal of data fusion is accurate data reconstruction, e.g., missing data estimation or link prediction, then identification of shared/unshared factors is not a major concern. On the other hand, in many applications, the goal of data fusion is to extract and interpret the underlying factors. For instance, in metabolomics applications, underlying factors need to be captured uniquely so that they can be used further to understand the patterns corresponding to a problem of interest, e.g., a specific type of diet or a disease. Therefore, in this paper, we develop a novel unsupervised data fusion model for joint factorization of heterogeneous data sets, which is quite effective in terms of revealing shared and unshared components. Using numerical experiments, we demonstrate that while the traditional formulation, modeling only shared factors, fails to capture the underlying structures in the presence of both shared and unshared components, the proposed model achieves accurate identification of shared and unshared components. Furthermore, we study a set of mixtures of known chemical composition by two analytical techniques, i.e., LC-MS and diffusion NMR. While NMR can capture all chemicals, one of the chemicals is invisible to LC-MS. We demonstrate the effectiveness of our model on this prototypical example using real data, where coupled data sets have both shared and unshared components. This is an extended version of our work [25] where, we have introduced our model briefly and discussed preliminary findings in cancer metabolomics. Here, we study the performance of the model in depth using both simulated and real data sets, where the underlying ground truth is known. Several other studies have also previously discussed methods revealing shared and unshared components. However, these studies either focus on coupled matrix factorizations [1, 2, 13, 26–29] or assume that the number of shared/unshared factors is pre-determined by the user based on the performance of joint factorization in the training set (when considered in a supervised setting) [30]. Modeling shared and unshared components has also been considered within the context of Canonical Correlation Analysis [31–34] focusing only on joint analysis of matrices.

We survey the related work further in Section “Related work”. In Section “Methods”, we introduce our data fusion model and the algorithmic approach. Section “Results and discussion” demonstrates the performance of the proposed approach on simulated and real data sets. Section “Conclusions” concludes with future research directions.

## Related work

Data fusion has been studied for decades dating back to the models aiming to capture the common variation in two data sets, i.e., Canonical Correlation Analysis [35]. Earlier studies on data fusion have focused on joint factorization of multiple matrices [1, 4, 8–12, 36–38]. The coupled matrix factorization problem is typically formulated as
1

where  and  are matrices coupled in the first mode/dimension and the factor matrix corresponding to the common mode, , is shared by both factorizations. Here, *R* indicates the number of factors. This formulation extends to factorization of multiple matrices coupled in different modes. In some applications such as in metabolomics, sparsity-inducing penalty terms are added to coupled matrix factorizations in order to extract interpretable factors [5, 39]. Recently, a convex formulation of coupled matrix factorizations has also been proposed [40]. Tensor factorizations [41–43] can also be considered as one way of jointly analyzing multiple matrices forming the slices of a third-order tensor; however, neither coupled matrix factorization nor tensor factorization methods can handle joint analysis of heterogeneous data sets.

As an extension of Eq. (1), joint factorization of heterogeneous data, e.g., a third-order tensor , coupled with a matrix , can be formulated as
2

where tensor  and matrix **Y** are simultaneously factorized through the minimization of the objective function in Eq. (2), which fits a CANDECOMP/PARAFAC (CP) [44, 45] model to  and factorizes **Y** in such a way that the factor matrix corresponding the common mode, i.e.,  is the same.  and  are factor matrices corresponding to the second and third modes of , respectively. We use the notation  to denote the CP model.  is the factor matrix that corresponds to the second mode of **Y**. This formulation of coupled matrix and tensor factorizations (CMTF), dating back to the studies of Harshman and Lundy [46] and Smilde et al. [16], has recently been a topic of interest in many studies [3, 14, 47–50]. The model has been extended to different loss functions [17, 22, 23], and tensor factorizations other than CP [17, 22, 50, 51]. It has also shown to be quite effective in addressing missing data estimation [24, 51, 52] and link prediction problems [22].

## Methods

### Model: structure-revealing coupled matrix and tensor factorizations

**Figure 2:**
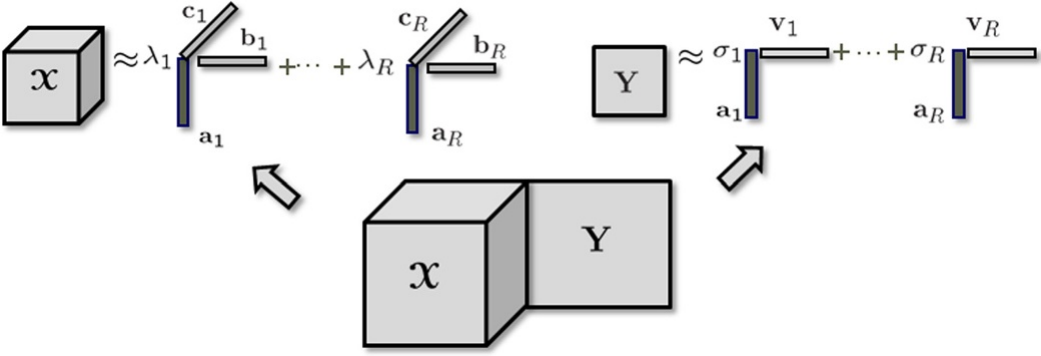
**Illustration of a coupled factorization of a third-order tensor and a matrix.**

The coupled matrix and tensor factorization model given in Eq. (2) makes an implicit assumption that all columns of factor matrix **A**, i.e., **a**_*r*_ for *r* = 1,…,*R*, are shared by the matrix and the third-order tensor, where *R* indicates the number of factors. When all factors are shared across data sets, the model can accurately capture the underlying factors [14]. However, in general, there are both shared and unshared factors in coupled data sets. Therefore, we reformulate the problem in such a way that through modeling constraints, we let the model identify shared/unshared components. We modify the objective function in Eq. (2) and rewrite the optimization problem as follows:
3

where  and  correspond to the weights of rank-one components in the third-order tensor and the matrix, respectively (Figure 2).  is a diagonal matrix with entries of *σ* on the diagonal. ‖. ‖ denotes the Frobenius norm for higher-order tensors/matrices and the 2-norm for vectors while ‖. ‖_1_ denotes the 1-norm of a vector, i.e., . *β* ≥ 0 is a penalty parameter. **a**_*r*_ denotes the *r*th column of **A**. In this formulation, our goal is to sparsify the weights *λ* and *σ* using the 1-norm penalties so that unshared components will have weights equal or close to 0 in one of the data sets.

In order to solve this constrained optimization problem, we first convert it into a differentiable unconstrained optimization problem and then use a first-order optimization algorithm. Using the quadratic penalty method [53], we convert the constraints into penalty terms. In order to make the objective function differentiable, we also replace the 1-norm terms with differentiable approximations, e.g., for sufficiently small *ε* > 0,  [54]. Our objective function can be formulated as follows, for *α*≥0:
4

#### Missing data

The model in Eq. (4) extends to joint analysis of incomplete data sets, i.e., data sets with missing entries. Missing data is encountered in many applications due to errors in the data collection process or costly experiments. In the presence of missing entries, we can still jointly analyze incomplete data sets by ignoring missing entries and modeling only the known data entries as follows:
5

where ∗ denotes the Hadamard product and  indicates the missing entries of  such that


Similarly,  indicates the missing entries in . Modeling only the known data entries has shown to be useful when fitting CP models in terms of both missing data estimation performance [55, 56] and computational efficiency [56]. Recently, we have also studied the CMTF model in Eq. (2) in terms of missing data estimation using a similar formulation [52]. Here, we only show that the structure-revealing CMTF model can easily handle missing data but we do not focus on the missing data estimation problem in this paper.

### Algorithm

Previously, we have studied the minimization of the objective for the original CMTF model in Eq. (2) [14] using an all-at-once gradient-based optimization approach, which solves the optimization problem for all factor matrices simultaneously. Here, we extend that work to fit the structure-revealing CMTF model and focus on the minimization of the objective function in Eq. (4).

We first briefly discuss the computation of the gradient. The gradient can be computed by taking the partial derivates of *f* with respect to the factor matrices and the vectors *λ* and *σ*. The gradient ∇*f* of size *R* (*I* + *J* + *K* + *M* + 2) can be formed by vectorizing the partials with respect to the factor matrices and concatenating them with the partials with respect to the vectors *λ* and *σ* as follows:


Let  and **Z** = **AΣV**^*T*^. Assuming that each term in *f* is multiplied by  for ease of computation, the partial derivatives can be computed as


where ×_*n*_ denotes the tensor-vector product in the *n*th mode; ⊙ denotes the Khatri-Rao product, and **X**_(*n*)_ denotes the tensor  unfolded in the *n*th mode. Unfolding (or matricization) in the *n*th mode rearranges a higher-order tensor as a matrix by using the mode-*n* fibers as the columns of the resulting matrix (See [42, 43] for details.)  corresponds to **A** with columns divided by their 2-norms. Here, *ε* is set to 10^-8^.

Once the gradient is computed, we use the Nonlinear Conjugate Gradient (NCG) method [53] with the Moré-Thuente line search as implemented in the Poblano Toolbox [57] (for the convergence properties of NCG, we refer interested readers to [53]). Any other first-order method such as the other algorithms implemented in the Poblano Toolbox can also be used to fit the model. Note that we are solving a non-convex optimization problem and cannot guarantee to reach the global minimum. Therefore, we use random initializations and pick the solution with the minimum function value in our experiments in the next section. The computational cost *per iteration* depends on the gradient computations, and in the case of a third-order tensor of size *N* × *N* × *N* coupled with a matrix of size *N* × *N*, the leading term in the gradient computation is *O* (*N*^3^*R*) for an *R*-component model.

## Results and discussion

In this section, we first compare the performance of our model with the traditional CMTF model using simulated coupled data sets in terms of identifying shared/unshared components. We then use the proposed model to jointly analyze LC-MS and NMR measurements of a set of mixtures with known chemical composition and demonstrate that our model can successfully capture the chemicals used in the mixtures, extract the relative concentrations of the chemicals accurately and provide promising results in terms of identifying shared/unshared chemicals.

### Simulations

We generate simulated coupled data sets with different underlying structures and compare the original CMTF formulation in Eq. (2) with the model in Eq. (4).

#### Experimental set-up

We generate factor matrices  and  with entries randomly drawn from the standard normal distribution. The columns of factor matrices are normalized to unit norm. Here, we set *I*=50,*J* = 30,*K* = 40 and *M* = 20. The factor matrices are used to construct a third-order tensor  coupled with matrix **Y** = **AΣV**^*T*^, where *λ* and diagonal entries of diagonal matrix **Σ**, i.e., *σ* of length *R*, correspond to the weights of rank-one third-order tensors and matrices, respectively. A small amount of Gaussian noise is added to data sets. Using four sets of weights, we generate cases where *R* components are shared differently among coupled data sets: (i) Case 1: One shared and one unshared component in each data set, i.e., **λ** = [ 1 0 1]^*T*^ and **σ** = [ 1 1 0]^*T*^, where *R* =3. (ii) Case 2: One unshared component in the matrix, i.e., **λ** = [ 1 1 0]^*T*^ and **σ** = [ 1 1 1]^*T*^, where *R* =3. (iii) Case 3: One unshared component in the third-order tensor, i.e., **λ** = [ 1 1 1]^*T*^ and **σ** = [ 1 1 0]^*T*^, where *R* =3. (iv) Case 4: One shared and one unshared component in the third-order tensor as well as two unshared components in the matrix, i.e., **λ** = [ 1 1 0 0]^*T*^ and **σ** = [ 1 0 1 1]^*T*^, where *R* = 4.

Once coupled data sets are generated, they are jointly factorized using the traditional CMTF model in Eq. (2) and our proposed structure-revealing CMTF model in Eq. (4) (referred to as Advanced CMTF (ACMTF)). As described in Section “Methods”, we use a gradient-based all-at-once optimization approach for fitting ACMTF, which we call ACMTF-OPT. Similarly, for fitting the model in Eq. (2), CMTF-OPT [14] is used and it is also based on a gradient-based all-at-once approach. Both CMTF-OPT and ACMTF-OPT are implemented in the MATLAB CMTF Toolbox (available from http://www.models.life.ku.dk). As stopping conditions, both methods use the relative change in function value (set to 10^-10^) and the 2-norm of the gradient divided by the number of entries in the gradient (set to 10^-10^).

#### Numerical results

**Figure 3:**
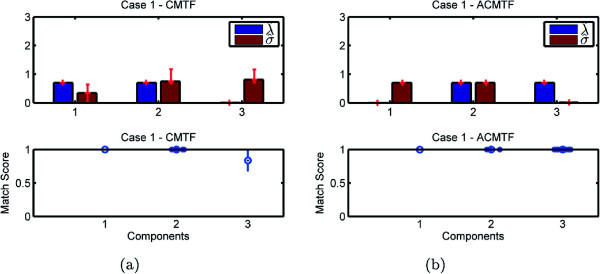
**Case 1 - Weights** ***λ*** **and** ***σ*** **as well as the match score for factor matrix A captured by (a) CMTF (b) ACMTF.**

**Figure 4:**
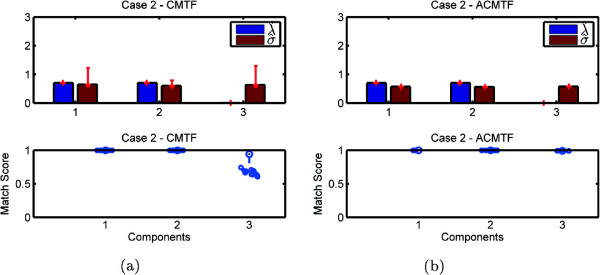
**Case 2 - Weights** ***λ*** **and** ***σ*** **as well as the match score for factor matrix A captured by (a) CMTF (b) ACMTF.**

**Figure 5:**
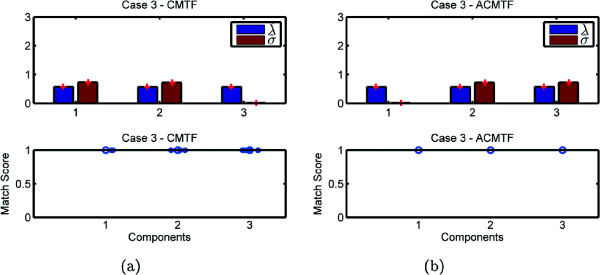
**Case 3 - Weights** ***λ*** **and** ***σ*** **as well as the match score for factor matrix A captured by (a) CMTF (b) ACMTF.**

**Figure 6:**
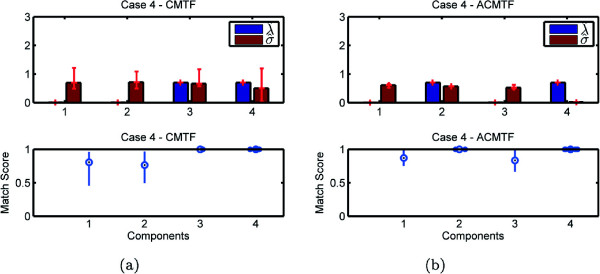
**Case 4 - Weights** ***λ*** **and** ***σ*** **as well as the match score for factor matrix A captured by (a) CMTF (b) ACMTF.**

Experiments demonstrate the potential problem with the CMTF model and how it fails to identify shared and unshared components due to uniqueness issues. On the other hand, our structure-revealing model can successfully identify shared/unshared components through the use of sparsity penalties on the component weights. Figures 3, 4, 5, and 6 demonstrate the weights, ***λ*** and ***σ***, estimated using both models for 100 runs returning the same function value^a^, i.e., multiple random starts are used and the minimum function value is obtained 100 times. When we use CMTF, ***λ*** and ***σ*** are estimated by normalizing the columns of the extracted factor matrices. In Figure 3, we expect to recover ***λ***= [ 1 0 1]^*T*^ and ***σ***= [ 1 1 0]^*T*^; however, we observe that weights captured by CMTF vary hiding the true underlying structure of the data sets. On the other hand, ACMTF reveals the true structure indicating that there is one shared and one unshared component in each data set. The order of original and extracted components is different due to the permutation ambiguity in the models. Also, due to the permutation ambiguity, all possible permutations of the components for different runs returning the minimum function value are compared and the results are reported based on the best matching permutation^b^. Bottom plots in Figure 3 show how well the extracted factors match with the true columns of factor matrix *A*. Let  be the *r*th column of the factor matrix  extracted from the common mode. The match score corresponds to  after finding the best matching permutation of the columns. These plots show that not only the weights can indicate shared/unshared components but also factor vectors can be estimated well using ACMTF. Similarly, in Figure 4, we expect to see three non-zero weights for the matrix and two non-zero weights for the tensor. However, there is variation for the same function value particularly in *σ* hiding the structure of the data sets and preventing recovery of the factor vectors accurately when data sets are modeled using CMTF. ACMTF, on the other hand, can identify shared and unshared components accurately. Unlike Case 1 and 2, CMTF performs well for Case 3, where the tensor has all three components and two of them are shared with the matrix (Figure 5).

While ACMTF performs well for all three cases, we should note that uniqueness properties of the model need to be better understood. For instance, in Case 4, there are two unshared components in the matrix and, in Figure 6, match scores for ACMTF indicate that underlying factors can no longer be perfectly recovered. That is mainly because the model is no longer unique. Two unshared components in the matrix span the same subspace in different runs returning the same function value but components from different runs can no longer be compared using the match score.

**Figure 7:**
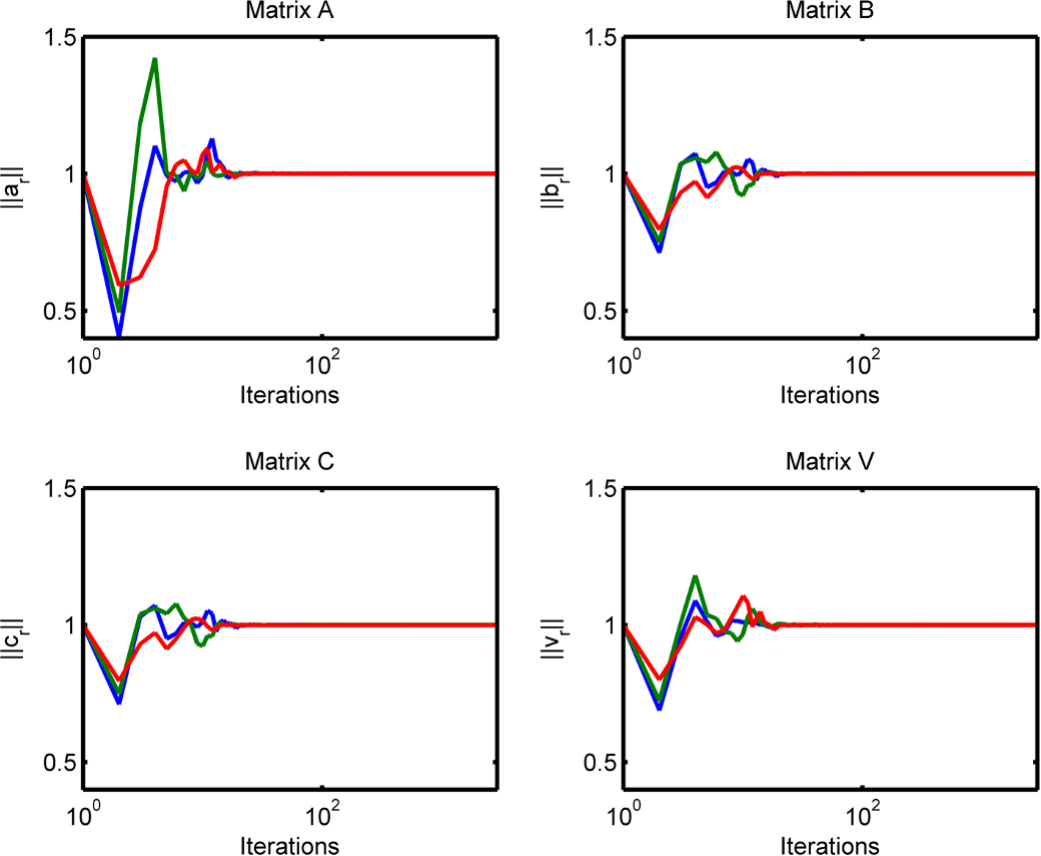
**2-norm of each column of the factor matrix in each mode.**

We also show how effective the penalty method is in terms of satisfying the unit-norm constraints in Figure 7. Figure 7 illustrates the 2-norm of each column of the factor matrix in each mode as the algorithm runs. We observe that while norms of the columns fluctuate initially, when the algorithm stops, they are all close to 1. This indicates that even though we solve the constrained optimization problem in (3) using the quadratic penalty method, we can still satisfy the constraints. The parameter *α* is set to *α* = 1 for all modes since we want the quadratic penalty terms to have the same weight as the first two terms in the objective in Eq. (4). Note that before fitting the model, each data set, i.e., tensor  and matrix **Y**, is divided by its Frobenius norm. Therefore, by selecting *α* = 1, we give equal importance to every term in the objective except the sparsity-inducing penalties. We use *β* = 10^-3^ as the sparsity penalty parameter in our experiments.

**Figure 8:**
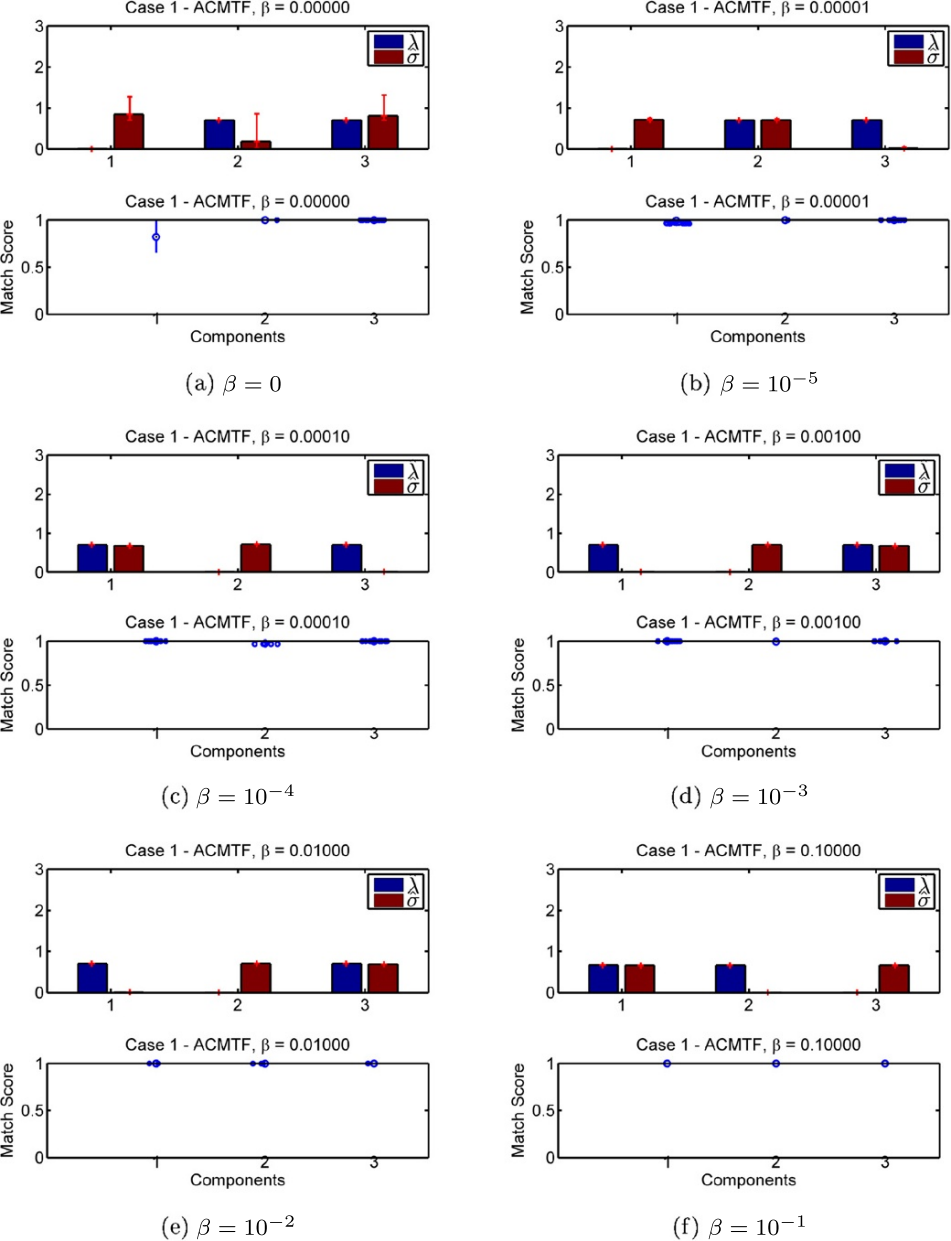
**Sensitivity of ACMTF with respect to** ***β*** **.**

In order to assess the sensivity of ACMTF to the selection of the *β* value, we show the performance of the model for Case 1 using different *β* values, i.e, *β* ∈ {0,10^-5^,10^-4^,10^-3^,10^-2^,10^-1^} in Figure 8. We observe that except for *β* = 0, shared and unshared factors can be correctly identified for all other *β* values. However, for higher values of *β*, i.e., *β* = 10^-2^ and *β* = 10^-1^, it becomes difficult to get the true solution, i.e., out of 1000 random starts, only few runs return the true solution for high *β* values while the true solution is reached by approximately 50%–75% of the random starts for *β* = 10^-4^ or *β* = 10^-5^^c^.

Finally, we discuss how we interpret the extracted weights. For instance, for Case 1, while the true nonzero weights are set to 1 in *λ* and *σ* when generating the data sets, the estimated values of the nonzero weights by the ACMTF model are approximately 0.70 in Figure 3(b). That is due to the fact that models are fitted to data sets divided by their Frobenius norms, which are approximately 1.42. In order to find the actual weights in each data set, we would multiply the captured weights by the norm of each data set. However, in joint data analysis, we are looking for weights that can show the relative significance of a factor in one data set with respect to the other data sets, rather than absolute weights in each data set. For instance, if we generate coupled data sets using **λ**= [ 100 0 100]^*T*^ and **σ** = [ 1 1 0]^*T*^, the ACMTF model still reveals the weights given in Figure 3(b). Furthermore, if a factor has different contributions to the data sets, that will also be revealed by the weights. For instance, in Case 2, data sets are generated using **λ** = [ 1 1 0]^*T*^ and **σ** = [ 1 1 1]^*T*^, where the shared component contributes more to  compared to **Y**. That is revealed by the weights extracted by the ACMTF model in Figure 4(b), where  and .

#### Extension to multiple data sets

**Figure 9:**
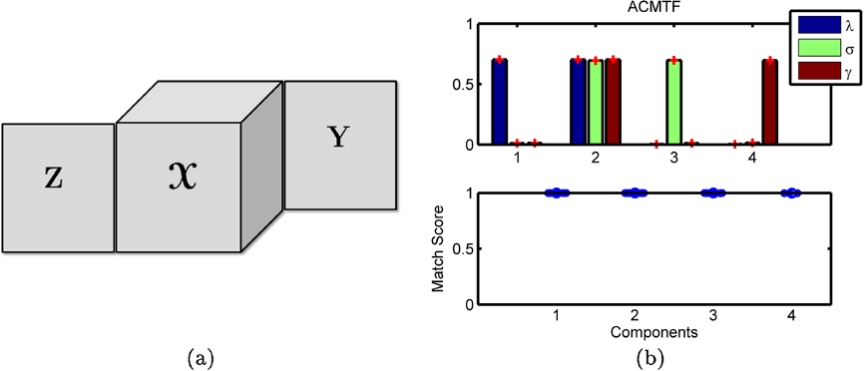
**Modeling of more than two data sets using ACMTF.** **(a)** A third-order tensor  coupled with matrices **Y** and **Z** in the first mode, **(b)** Weights ***λ,σ***and ***γ***captured by ACMTF as well as the match score for factor matrix **A**.

Our experiments so far have focused on joint analysis of two data sets. Here, we also demonstrate that the proposed model has a promising performance in terms of identifying shared/unshared factors when more than two data sets are jointly analyzed. We use the coupled data sets given in Figure 9(a) as an illustrative example.

In order to construct the data sets in Figure 9(a), factor matrices ,  and  are generated as described in the Experimental set-up section. Here, we set *I* = 50,*J* = 30,*K* = 40, *M* = 20,*L* = 40, and *R* = 4. Factor matrices are then used to construct a third-order tensor  coupled with **Y** = **AΣV**^*T*^and **Z** = **AΓS**^*T*^in the first mode, where *λ*, diagonal entries of the diagonal matrix **Σ**, i.e., *σ*, and diagonal entries of the diagonal matrix **Γ**, i.e., **γ**, correspond to the weights of the components. Figure 9(b) demonstrates the performance of the ACMTF model in terms of identifying shared/unshared components when each data set has one shared and one unshared component; in other words, data sets are generated using the weights **λ** = [ 1 1 0 0]^*T*^, **σ** = [ 1 0 1 0]^*T*^, and **γ** = [ 1 0 0 1]^*T*^. We observe that the extracted weights reveal that there is one component shared by all three data sets and one unshared component in each data set.

### Real data

In this section, the structure-revealing CMTF model is used to jointly analyze diffusion NMR and LC-MS measurements of 29 mixtures prepared using five chemicals. We first describe the sample preparation and the measurements, and then demonstrate the performance of our model in terms of capturing the signatures/patterns related to chemicals used to prepare the mixtures.

#### Sample preparation and measurements

Five chemicals with different relative sizes, hence, different diffusion, were selected: two peptides, a single amino acid, a sugar and an alcohol, i.e., Valine-Tyrosine-Valine (Val-Tyr-Val), Tryptophan-Glycine (Trp-Gly), Phenylalanine (Phe), Maltoheptaose (Malto) and Propanol. 29 samples were prepared with varying concentrations according to a predetermined design (see Additional file 1) in a phosphate buffer (pH 7.4). The buffer was prepared with deuterated water according to a protocol for biological samples [58] but with a 10-fold increase in the concentration of TSP (sodium 3-(trimethylsilyl)-propionate-2,2,3,3-d _4_) in order to ensure sufficient signal intensity for reference deconvolution [59]. The 10-fold increase in the concentration of TSP did not affect the pH of the buffer. All chemicals were purchased from Sigma Aldrich and used without further purification. Samples were stored at 5°C until they were measured.

NMR spectra of the samples were recorded on a Bruker DRX 500 spectrometer (Bruker Biospin Gmbh, Rheinstetten, Germany) operating at a proton frequency of 500.13 MHz. For each spectrum, 32768 complex points were acquired in 64 scans with a recycle delay of 2 seconds at a nominal temperature of 298 K. The spectrometer was equipped with a 5 mm BBI probe and spectra were recorded using the Oneshot45 sequence [60] with 8 gradient levels ranging from 3.4 to 26.9 G cm ^-1^ with equal steps in gradient squared in nominal gradient amplitude. The diffusion time was 100 ms and the gradient encoding time was 1 ms. All processing of the data, including phase correction, apodization, Fourier transformation, baseline correction, referencing to TSP signal, and reference deconvolution, was performed using the DOSY Toolbox [61]. In order to correct for instrument instabilities, reference deconvolution was performed using the TSP methyl signal as a reference, using a target lineshape of 4.5 Hz [59, 62]. The MATLAB code for the DOSY toolbox is freely available via http://dosytoolbox.chemistry.manchester.ac.uk/. NMR measurements for each mixture correspond to a set of spectra recorded at different gradient levels. Since we have several mixtures, NMR data can be arranged as a third-order tensor with modes: mixtures, chemical shift and gradient levels (Figure 1). The chemical shift (i.e., the conventional scale for a ^1^H NMR spectrum) is related to the chemical environment of the protons, and the gradient levels encode the diffusion property of the various molecular species.

Prior to LC-MS measurements, 29 samples were diluted to 10 ppm in water and subsequently analyzed with ultra-performance liquid chromatography (UPLC) system coupled to quadruple time-of-flight (Premier QTOF) mass spectrometer (Waters Corporation, Manchester, UK). Each sample (10*μL*) was injected into the UPLC equipped with a 1.7*μm* C18 BEH column (Waters) operated with a 6-min linear gradient from 0.1*%* formic acid in water to 0.1*%* formic acid in 20*%* acetone: 80*%* acetonitrile. The data were acquired on the positive electrospray ionization (ESI) mode with the following settings: capillary probe voltage was set to 2.8 keV, desolvation gas temperature was at 400°C, cone voltage was 40 V, with the Ar collision gas energy of 10 V. The centroided raw data were converted to an intermediate netCDF format with the DataBridge ^*TM*^ utility provided with the MassLynx software. Automatic peak detection and integration were performed using the XCMS package [63]. Since individual chemical compounds give rise to more than one fragment ion upon ionization, these ion-features, generated by XCMS, were grouped together using the CAMERA package [64]. The final LC-MS data set is in form of a mixtures by features matrix (Figure 1).

#### Analysis

**Figure 10:**
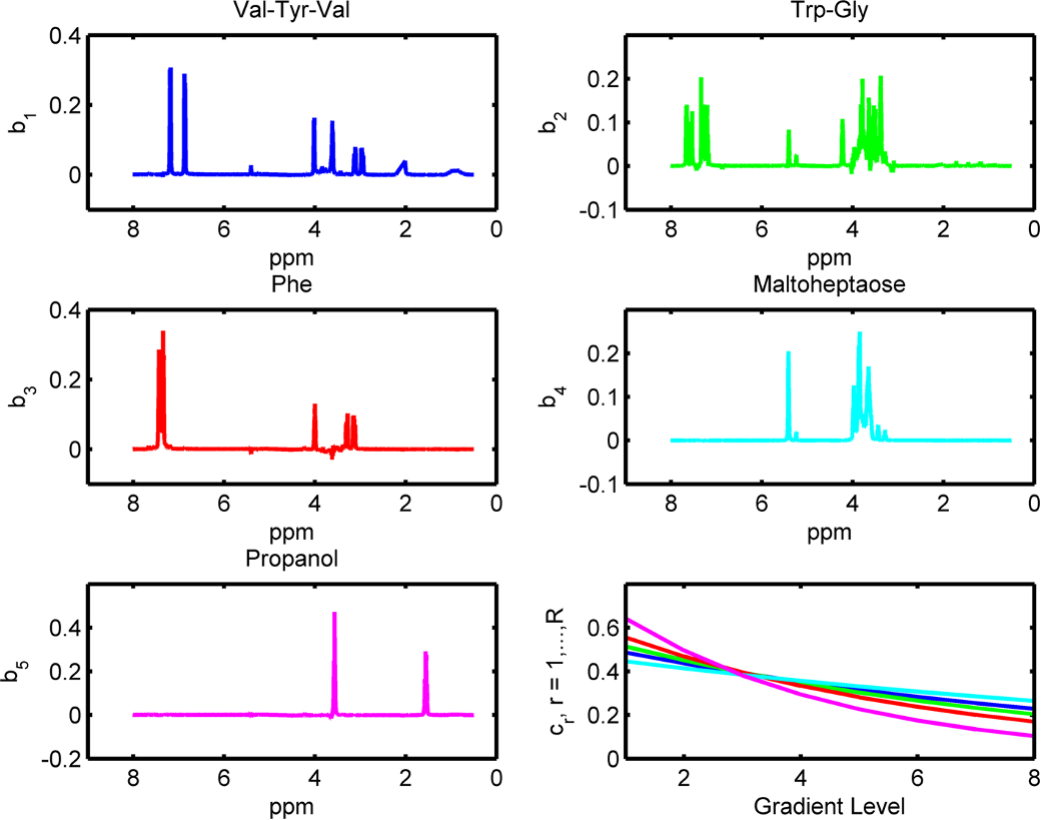
**Columns of factor matrix B corresponding to the chemical shift (ppm) mode (i.e., NMR spectra).** The figure in the bottom-right corner shows the columns of factor matrix **C** corresponding to the gradient levels mode. These are the factor matrices captured by the CP model of NMR data.

**Figure 11:**
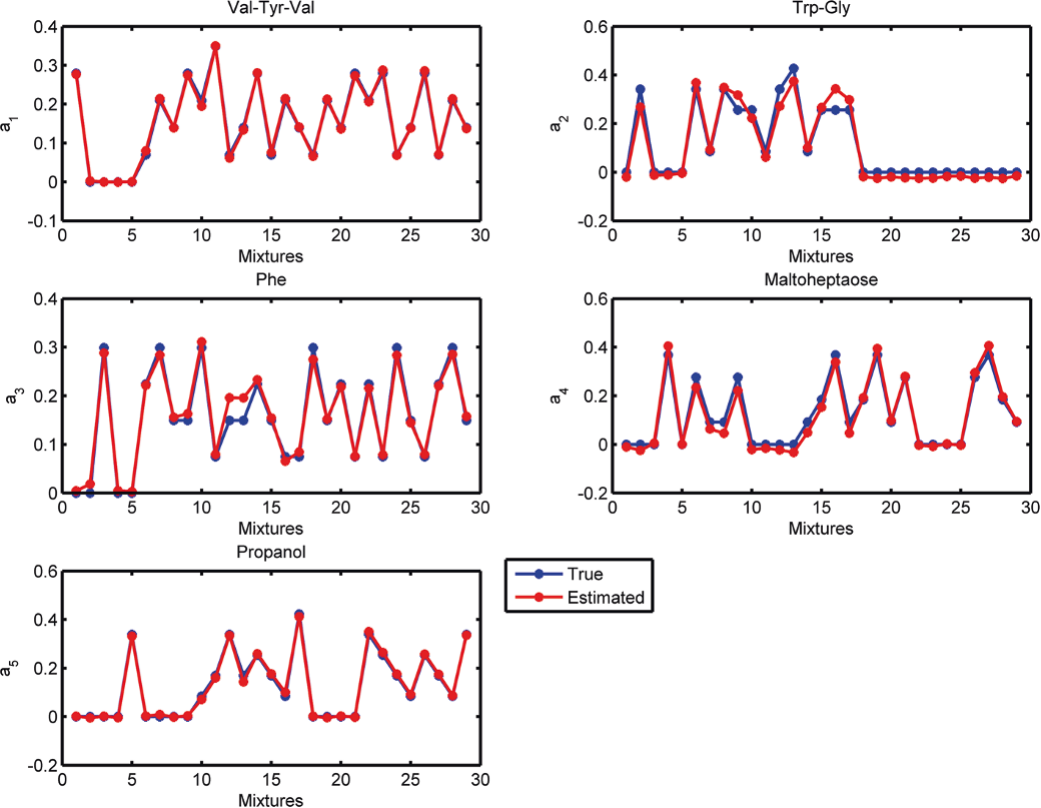
**Columns of factor matrix A corresponding to the mixtures mode extracted by the CP model of NMR data.** Red lines show the columns of **A** while the blue line shows the original relative concentrations of the chemicals used in sample preparation, i.e., normalized columns of the matrix given in Additional file 1.

Before discussing joint analysis of the third-order tensor  representing diffusion NMR measurements and the matrix **Y** representing LC-MS data (Figure 1), we briefly discuss the analysis of the NMR data individually.  has an underlying CP structure [20, 21, 65–68] and can be modeled using a CP model, i.e., . Here, **A**,**B** and **C** correspond to the factor matrices in the mixtures, chemical shift and gradient levels modes, respectively. When we model  using a 5-component CP model, we observe that each CP component corresponds to one of the chemicals used in the mixtures. The signatures in the chemical shift mode (the NMR spectra), i.e., the columns of matrix **B**, as well as the exponential decay signatures represented by the columns of matrix **C** can be used to identify these chemicals. Figure 10 shows the NMR signatures extracted by the CP model (Signatures in the chemical shift mode (spectra) of pure chemicals are given in Additional file 2 as a reference). Matrix **A** captures the relative concentrations of the extracted components in the mixtures and we observe that **A** matches well with the design used in sample preparation in Figure 11. Matrix **Y** representing LC-MS measurements can be analyzed individually using matrix factorizations. However, matrix factorizations without any constraints on the factors have a rotational freedom; therefore, capturing the patterns corresponding to each chemical using only LC-MS data is challenging. One potential approach may be to use sparse principal component analysis [69]; however, even with careful fine-tuning of the sparsity parameter, the underlying design cannot be captured as well as in Figure 11 due to unavoidable experimental noise in LC-MS (results not shown).

Analysis of the diffusion NMR data not only reveals the underlying structures in the chemical mixtures but can also be used to extract the relevant patterns corresponding to the same chemicals from data sets, which are much harder to analyze, e.g., LC-MS measurements. LC-MS data are often noisy and contain many irrelevant features due to the sensitivity of the analytical technique. Next, we jointly analyze NMR and LC-MS measurements using the structure-revealing CMTF model and demonstrate the benefits of joint analysis of these data sets. As a preprocessing step, LC-MS features are scaled by their standard deviations and both NMR and LC-MS data sets are scaled by their respective Frobenius norms. We jointly analyze the data sets using (i) Model 1: ACMTF model with no sparsity penalty, i.e., *β* = 0, and (ii) Model 2: ACMTF model with sparsity penalties on the weights of rank-one components, where *β* = 10^-3^. For both models, the number of components is set to *R* = 6. Since there are five chemicals in the samples and we expect to have some experimental noise, we use *R*=6 components. We discuss the choice of the number of components further in the Discussion section.

**Figure 12:**
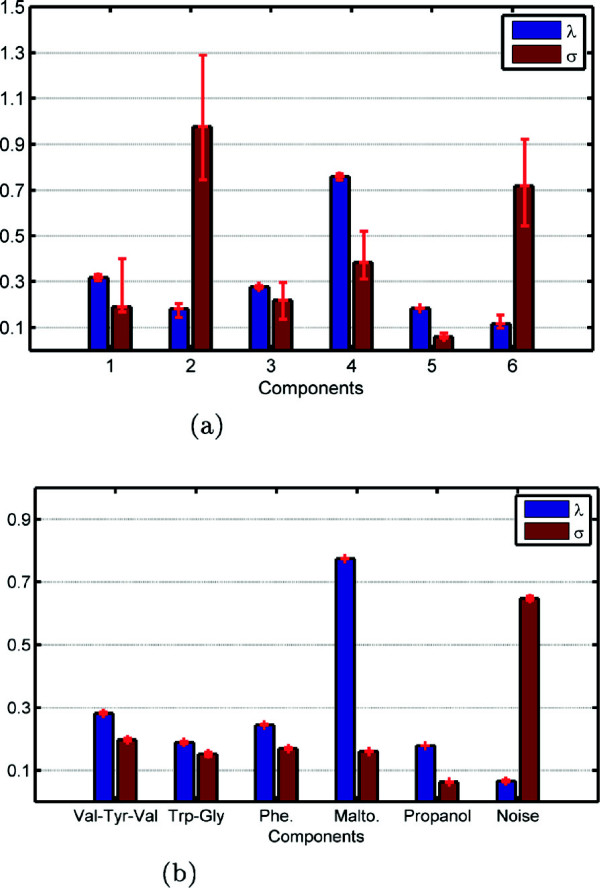
**Weights** ***λ*** **and** ***σ*** **captured by (a) Model 1 (** ***β*** **= 0) and (b) Model 2 (** ***β*** **= 10** ^**-3**^ **).**

**Figure 13:**
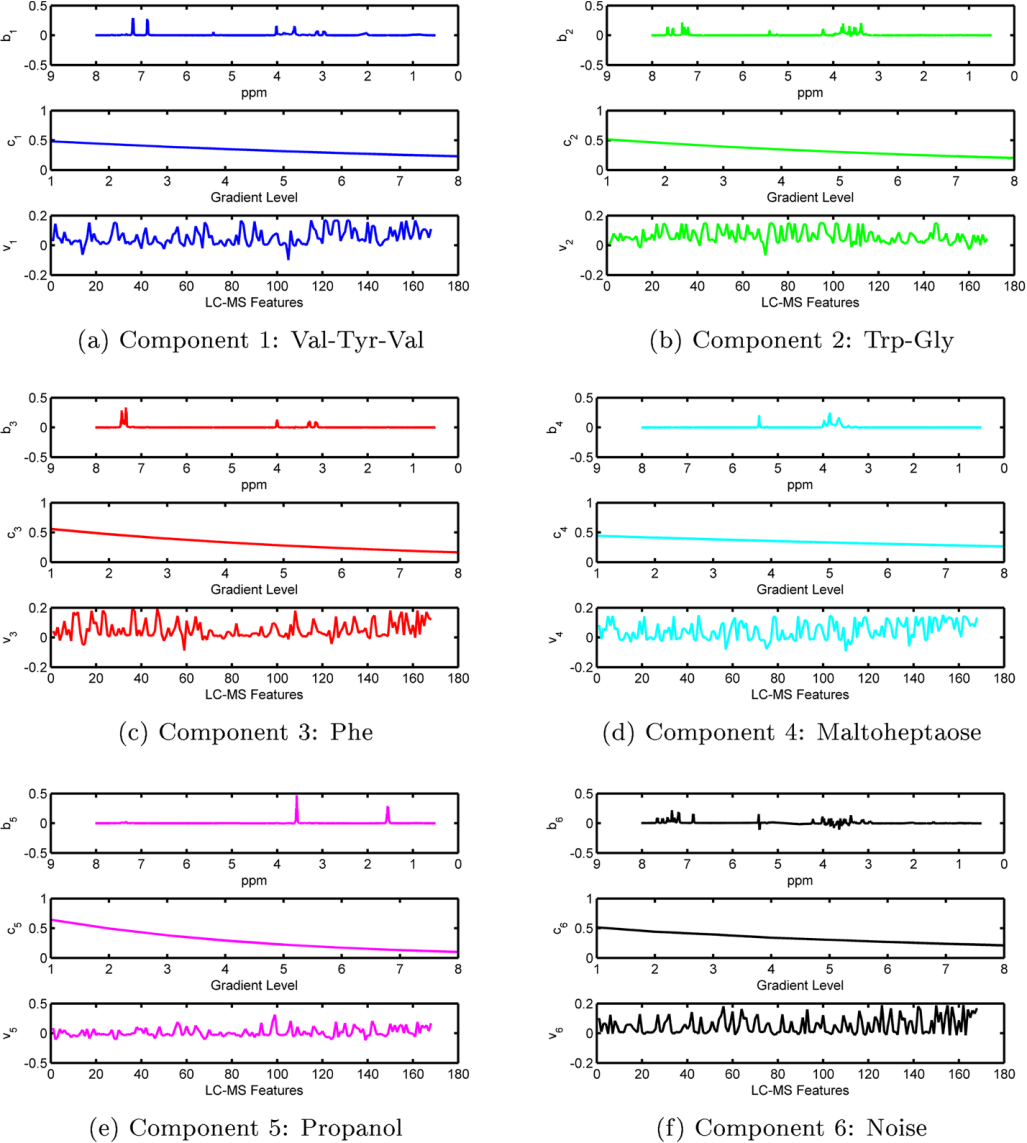
**Model 2 - Components extracted by coupled factorization of NMR and LC-MS using ACMTF, where** ***β*** **= 10** ^**-3**^ **.** Columns of factor matrices **B, C** and **V** are plotted.

**Figure 14:**
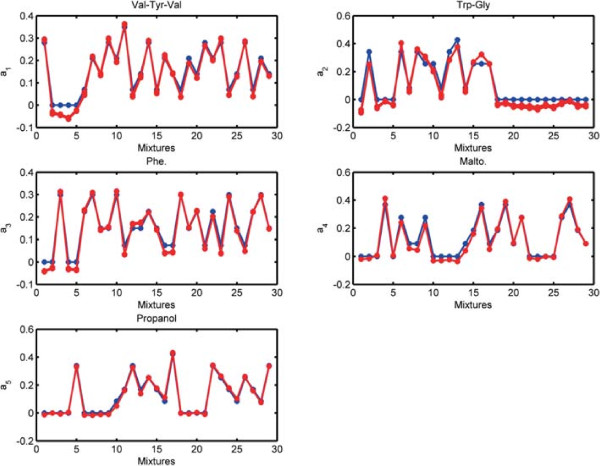
**Model 2 - Scores.** Columns of factor matrix **A** corresponding to the mixtures mode captured by coupled factorization of NMR and LC-MS data using ACMTF, where *β* = 10^-3^. Red lines show the columns of **A** while the blue line shows the original relative concentrations of the chemicals in mixtures, i.e., normalized columns of the matrix given in Additional file 1.

Model 1 is equivalent to the traditional CMTF model in the sense that it does not impose sparsity on the weights of rank-one components. Similar to our observations on simulated data sets, we observe that weights captured by Model 1 (Figure 12(a)) for the runs returning the same function value suggest that the model fails to give a unique solution. Model 2, on the other hand, captures the weights given in Figure 12(b) for the runs returning the same function value, which suggests uniqueness, and extracts the components illustrated in Figure 13. The model is able to capture the underlying chemicals and, as shown in Figure 14, it is also effective in terms of capturing the underlying design used in sample preparation. In Figure 14, we plot the columns of the factor matrix **A** for all (98) runs returning the same function value in red and the true design is plotted in blue. This further illustrates the suggested uniqueness of the model. In order to understand how components are shared among data sets, we look at the weights of rank-one components in Figure 12(b). While the components corresponding to Val-Tyr-Val, Trp-Gly, Phe and Malto are shared by both data sets, the component corresponding to propanol has a very small weight (<0.1) in LC-MS. Since propanol is not retained in the liquid chromatography column and eluted with the solvent front, it does not show up in LC-MS measurements; therefore, having a small weight for propanol in LC-MS is promising. Similarly, one of the components in LC-MS is modeling noise (which could be both structured and random) and barely shows up in NMR. That is also expected since this LC-MS data set is very noisy compared to the NMR data.

By individually analyzing NMR data, we have been able to capture NMR signatures of the chemicals. The benefit of jointly analyzing NMR and LC-MS, on the other hand, is two-fold: (i) In addition to the NMR signatures, we also extract the factor vectors corresponding to the LC-MS feature mode for each chemical as shown in Figure 13. The features with high coefficients (in terms of absolute value) in each factor reveal the features relevant to the chemical modeled by that component (see Additional file 3 for LC-MS features captured by the model for each component). (ii) Weights of rank-one components in each data set give an indication of the chemicals visible to each analytical technique.

#### Discussion

Even though the main motivation for a structure-revealing coupled factorization model is to identify shared/unshared components automatically through modeling constraints, there are still several parameters to be determined: (i) number of components (*R*) and (ii) sparsity penalty parameter (*β*). In order to see the sensivity of joint factorization of NMR and LC-MS to these parameters, we have fit the model using different *β* values, i.e., *β* ∈ {10^-4^,10^-3^,10^-2^,10^-1^}, for different number of components, i.e., *R* ∈ {5,6,7,8}. If we use *β* = 10^-4^ or *β* = 10^-2^, there are small variations in the weights captured by the runs returning the same function value even though the weights are close to what we have obtained in Model 2 using *β* = 10^-3^. Using a much higher *β* value, i.e., *β* = 10^-1^, on the other hand, sparsifies the weights introducing many zeros and fails to capture the underlying chemicals. In terms of the number of components, while the three-way NMR data set has 5 components, fitting a 5-component coupled model cannot find the underlying components accurately due to the additional structured/random noise in LC-MS. The singular values of the centered-scaled LC-MS data suggest that there are 6 significant components. Model 2, we have discussed so far, is a 6-component model but since we have not centered LC-MS data, we have also tried 7 and 8-component models. Using a 7-component model, true chemicals can still be captured but the additional component does not look meaningful and slightly distorts the true components. Using an 8-component model, we have similar observations except that the 8th component has a very small weight (<0.1) in both data sets indicating that we may be overfactoring the data. We plan to study and improve the robustness of the model to overfactoring, which can make it easier to choose the number of components.

In our analysis, we have downsampled the NMR spectra by a factor of 10 because we use many random starts to find the “true” solution and it is more efficient to work with downsampled NMR data. However, for better interpretability of NMR spectra, high digitization is needed. When we jointly analyze LC-MS data with the original NMR data, which have not been downsampled, using the same model parameters used for Model 2, the model reveals almost exactly the same components and weights, showing that the model is not sensitive to minor changes in the data.

While the model is promising, we should note that it is not perfect even for simple mixtures like we have analyzed here. One of the issues is that columns of factor matrix **V** corresponding to the LC-MS features mode are dense and not easily-interpretable. The *r*th column of **V** contains features corresponding to the chemical which has its NMR signatures as the *r*th column of matrix **B** and **C**; however, in addition to the relevant features, it also contains irrelevant features regarded as false-positives (see Additional file 3). Another issue is that it would be more useful to get zero weights instead of small weights for unshared components (as in simulated data sets). As pointed out in Section “Background”, several methods have been proposed for the identification of shared/unshared components within the context of joint analysis of matrices, and the performance comparison of those methods with the structure-revealing CMTF model is a topic of future research. However, note that since these methods focus on joint analysis of matrices, there are identifiability issues and the identifiability of the models are achieved using constraints on the components, such as orthogonality in CCA-based approaches [34] and GSVD-based methods [1]. The structure-revealing CMTF model, on the other hand, does not impose any constraints on the components (other than the unit norm constraints). The structure-revealing CMTF model has such an advantange over joint matrix factorization methods because the CP model used to model the higher-order tensor is capable of uniquely capturing the underlying factors. The CP model is unique under mild conditions up to permutation and scaling (for a review of uniqueness studies, see [43]). Furthermore, while we have seen that the structure-revealing CMTF model extends to multiple data sets, some of these joint matrix factorization methods have only been proposed for two data sets [34].

#### Potential biological applications of interest

In this section, we have illustrated how the structure-revealing CMTF model can be used to capture chemicals in mixtures measured using different analytical methods. In order to study both the strengths and the limitations of the model, we have used prototypical experimental coupled data sets, where the underlying ground truth is known. In many biological applications, we come across with such heterogeneous coupled data sets. For instance, the potential of fluorescence spectroscopic measurements of human plasma samples in cancer diagnostics has recently been demonstrated, and based on the prior chemical knowledge, the fluorescence measurements are expected to follow a CP model [70]. In fluorescence spectroscopy, measurements for each sample are represented as an excitation-emission matrix, and multiple samples form a third-order tensor with modes: samples, excitation and emission wavelengths. Plasma samples can also be measured using LC-MS and NMR, which are commonly used in metabolomics studies [6]. Measurements from LC-MS and NMR are usually arranged as samples by features matrices. In a recent study [25], we have jointly analyzed fluorescence and NMR measurements of plasma samples of a group of verified colorectal cancer patients and a group of controls with nonmalignant findings using the structure-revealing CMTF model. The preliminary results demonstrate that there are shared/unshared components, and two of the shared components achieve around 71.4*%* accuracy (with 63.6*%* sensitivity and 78.1*%* specificity) in terms of separating the two groups. Even though the number of chemicals that can be detected by fluorescence spectropscopy is limited compared to the chemicals detectable by NMR, the components extracted from the fluorescence data are easily interpretable, and this can make the identification of biomarkers easier.

Such heterogeneous coupled data sets are also encountered in biomedical signal processing. In order to have a better understanding of brain activities, it is highly desirable to jointly analyze EEG (electroencephalogram) and fMRI (functional Magnetic Resonance Imaging) signals because EEG has a high temporal resolution while fMRI provides a better spatial resolution. Current data fusion approaches for EEG and fMRI rely on joint analysis of fMRI data with signals from a single EEG channel or concatenated signals from multiple channels [71, 72]. On the other hand, it may be possible to arrange multi-channel EEG signals as a third-order tensor and jointly factorize the tensor with the matrix representing the fMRI data using the structure-revealing CMTF model [72].

## Conclusions

Joint analysis of data sets from multiple sources has the potential to enhance knowledge discovery. However, we are still lacking the data mining tools for data fusion and need a better understanding of the available models in order to improve them to address the challenges in data fusion. In this paper, we have introduced an unsupervised data fusion model that can jointly analyze heterogeneous, incomplete data sets with shared/unshared components by formulating data fusion as a coupled matrix and tensor factorization problem with sparsity penalties on the weights of rank-one components. Using numerical experiments, we have demonstrated that the proposed model outperforms the traditional coupled factorization model commonly used in the literature in terms of identifying shared/unshared components. Furthermore, we have measured a set of mixtures with known chemical composition using two different analytical techniques (LC-MS and NMR) and assessed the performance of the proposed model in terms of capturing the underlying chemicals, true design and shared/unshared components. The model provides promising performance and reveals the ground truth in these mixtures. In addition to the strengths of the proposed model, we have also discussed the potential drawbacks using this illustrative example.

While the structure-revealing CMTF model inherits uniqueness properties from the CP model, the overall uniqueness properties of the structure-revealing CMTF model need to be understood better, as it has been done for coupled CP factorizations in a recent study [73].

We intend to extend our studies in several directions: (i) In order to extract easily-interpretable patterns with less false-positives from LC-MS features mode, we plan to impose sparsity constraints on the factors. Our preliminary studies show that we can decrease the number of false-positives; however, the model distorts the NMR signatures. (ii) Our algorithmic approach based on unconstrained optimization is accurate but not flexible enough to impose constraints. The feasibility of a more flexible modeling framework for data fusion making use of general purpose optimization solvers will be explored in future studies [74].

## Endnotes

^a^ Function values are considered the same if they have all digits the same up to the sixth decimal place.

^b^ When we fit the models and obtain the same function value multiple times, the *i*th coupled component (**a**_*i*_,**b**_*i*_,**c**_*i*_,**v**_*i*_) in one run may be the *j*th coupled component (**a**_*j*_,**b**_*j*_,**c**_*j*_,**v**_*j*_) in another run. Therefore, all possible permutations of the coupled components for different runs are compared to find the best matching components across different runs.

^c^ This is valid when function values are considered to be the same when the difference between them is less than 10^-6^.

## Electronic supplementary material

Additional file 1: **True design.** (PDF 46 KB)

Additional file 2: **Reference NMR signals.** (PDF 117 KB)

Additional file 3: **LC-MS features.** (PDF 55 KB)
