# Supplementary material for: Structure-revealing data fusion
Source: BMC Bioinformatics. 2014 Jul 12;15(1):239. doi: 10.1186/1471-2105-15-239 (PMC4117975; doi:10.1186/1471-2105-15-239)
Supplement: Supplementary file 1 — Additional file 1: True design. (PDF 46 KB) [file 12859_2013_6517_MOESM1_ESM.pdf]

True Design

Concentrations of the chemicals showing the true design used in mixture preparation are given in Table 1.

Table 1: Concentrations of the chemicals in milliMolar.

Val-Tyr-Val	Trp-Gly	Phe	Malto	Propanol
5.00	0	0	0	0
0	5.00	0	0	0
0	0	5.00	0	0
0	0	0	5.00	0
0	0	0	0	5.00
1.25	5.00	3.75	3.75	0
3.75	1.25	5 .00	1.25	0
2.50	5.00	2.50	1.25	0
5.00	3.75	2.50	3.75	0
3.75	3.75	5.00	0	1.25
6.25	1.25	1.25	0	2.50
1.25	5.00	2.50	0	5.00
2.50	6.25	2.50	0	2.50
5.00	1.25	3.75	1.25	3.75
1.25	3.75	2.50	2.50	2.50
3.75	3.75	1.25	5.00	1.25
2.50	3.75	1.25	1.25	6.25
1.25	0	5.00	2.50	0
3.75	0	2.50	5.00	0
2.50	0	3.75	1.25	0
5.00	0	1.25	3.75	0
3.75	0	3.75	0	5.00
5.00	0	1.25	0	3.75
1.25	0	5.00	0	2.50
2.50	0	2.50	0	1.25
5.00	0	1.25	3.75	3.75
1.25	0	3.75	5.00	2.50
3.75	0	5.00	2.50	1.25
2.50	0	2.50	1.25	5.00
