# Supplementary material for: Structure-revealing data fusion
Source: BMC Bioinformatics. 2014 Jul 12;15(1):239. doi: 10.1186/1471-2105-15-239 (PMC4117975; doi:10.1186/1471-2105-15-239)
Supplement: Supplementary file 2 — Additional file 2: Reference NMR signals. (PDF 117 KB) [file 12859_2013_6517_MOESM2_ESM.pdf]

Reference NMR signals

The chemical shift mode signatures of pure chemicals are illustrated in Figure 1.

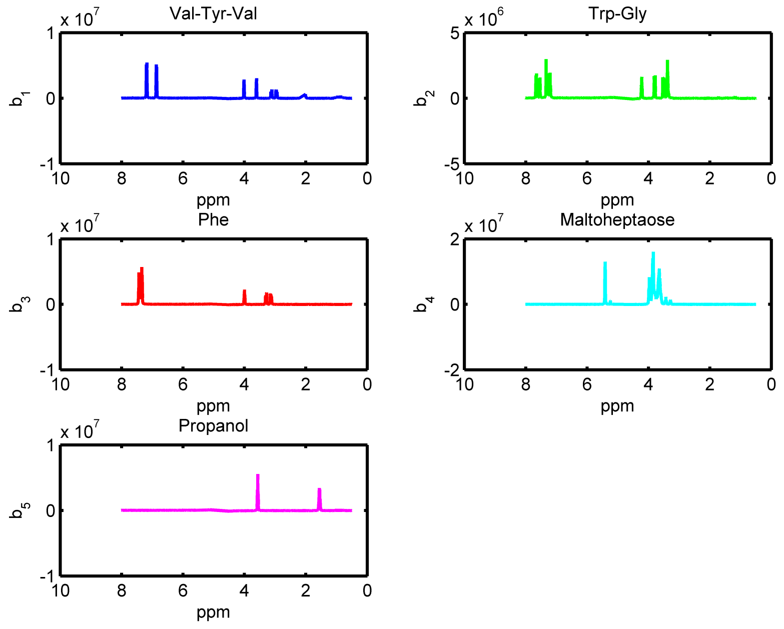

Figure 1: Chemical shift mode signatures of chemicals used in mixture preparation.
