# Supplementary material for: Structure-revealing data fusion
Source: BMC Bioinformatics. 2014 Jul 12;15(1):239. doi: 10.1186/1471-2105-15-239 (PMC4117975; doi:10.1186/1471-2105-15-239)
Supplement: Supplementary file 3 — Additional file 3: LC-MS features. (PDF 55 KB) [file 12859_2013_6517_MOESM3_ESM.pdf]

LC-MS Features

Table 1 shows the top 10 features in the factors extracted from LC-MS features mode by Model 2. The number of true features (out of 168 features) for each chemical is as follows: Val-Tyr-Val (14), Trp-Gly (16), Phe (12) and Malto (27).

Table 1: Top 10 features (in decreasing order based on the absolute value of their coefficients) in the factors extracted from LC-MS features mode.

	**Model 2**		
	**Mass Value**	**Retention Time**	
Val-Tyr-Val	380.22	1.70	TP
	420.25	1.71	TP
	362.21	1.70	TP
	392.92	0.59	**FP**
	759.43	1.69	TP
	263.14	1.70	TP
	702.87	0.54	**FP**
	394.23	1.93	**FP**
	421.26	1.70	TP
	388.28	1.67	**FP**
Trp-Gly	302.15	1.51	TP
	262.12	1.51	TP
	159.09	1.50	TP
	523.23	1.50	TP
	245.09	1.51	TP
	318.18	2.39	**FP**
	348.16	1.79	**FP**
	132.08	1.50	TP
	244.11	1.74	**FP**
	301.16	2.30	**FP**
Phe	166.09	1.42	TP
	149.06	1.42	TP
	131.05	1.42	TP
	770.85	0.54	**FP**
	566.89	0.54	**FP**
	150.07	1.42	TP
	120.08	1.42	TP
	331.17	1.42	TP
	634.88	0.54	**FP**
	906.83	0.54	**FP**
Malto	603.16	0.67	TP
	163.06	0.67	TP
	325.11	0.66	TP
	97.03	0.65	TP
	85.03	0.66	TP
	527.16	0.68	TP
	487.17	0.67	TP
	127.04	0.66	TP
	145.05	0.66	TP
	770.85	0.54	**FP**
